# Development of endomyocardial fibrosis model using a cell patterning technique: *In vitro* interaction of cell coculture of 3T3 fibroblasts and RL-14 cardiomyocytes

**DOI:** 10.1371/journal.pone.0229158

**Published:** 2020-02-24

**Authors:** Paola Orozco, Yuliet Montoya, John Bustamante

**Affiliations:** 1 Centro de Bioingeniería, Grupo de Dinámica Cardiovascular, Universidad Pontificia Bolivariana, Medellín, Colombia; 2 Comité de Trabajo de Bioingeniería Cardiovascular, Sociedad Colombiana de Cardiología y Cirugía Cardiovascular, Bogotá, Colombia; University of Cincinnati College of Medicine, UNITED STATES

## Abstract

Cardiac functions can be altered by changes in the microstructure of the heart, i.e., remodeling of the cardiac tissue, which may activate pathologies such as hypertrophy, dilation, or cardiac fibrosis. Cardiac fibrosis can develop due to an excessive deposition of extracellular matrix proteins, which are products of the activation of fibroblasts. In this context, the anatomical–histological change may interfere with the functioning of the cardiac tissue, which requires specialized cells for its operation. The purpose of the present study was to determine the cellular interactions and morphological changes in cocultures of 3T3 fibroblasts and RL-14 cardiomyocytes via the generation of a platform an *in vitro* model. For this purpose, a platform emulating the biological characteristics of endomyocardial fibrosis was generated using a cell patterning technique to study morphological cellular changes in compact and irregular patterns of fibrosis. It was found that cellular patterns emulating the geometrical distributions of endomyocardial fibrosis generated morphological changes after interaction of the RL-14 cardiomyocytes with the 3T3 fibroblasts. Through this study, it was possible to evaluate biological characteristics such as cell proliferation, adhesion, and spatial distribution, which are directly related to the type of emulated endomyocardial fibrosis. This research concluded that fibroblasts inhibited the proliferation of cardiomyocytes via their interaction with specific microarchitectures. This behavior is consistent with the histopathological distribution of cardiac fibrosis; therefore, the platform developed in this research could be useful for the *in vitro* assessment of cellular microdomains. This would allow for the experimental determination of interactions with drugs, substrates, or biomaterials within the engineering of cardiac tissues.

## Introduction

The mechanisms responsible for cardiac arrhythmias are grouped into two categories: impulse generation disorders and electrical conduction [1,2]. A pathology that produces alterations in the electrical conduction pathways is the endomyocardial fibrosis (EMF), from which microstructural and functional changes are derived. These changes lead to inadequate electrical conduction, which can result in slight alterations, or complete chaos in impulse transmission, which occurs in some arrhythmias, such as atrial fibrillations, ventricular fibrillations, and cardiac blocks [2–6].

Multifactorial processes are involved in EMF as a result of complex cellular interactions [7]. Among these processes, the proliferation of fibroblasts plays an important role in the production of extracellular matrix (ECM) proteins. Contrary to what happens in pathological conditions or tissue damage, these fibroblasts sustain the interstitial medium of the cardiomyocytes and regulate the supporting structure of the heart, actively participating in the healing process [8–11].

EMF arises as a result of the accumulation of collagen deposits in the ECM, as a consequence of the evolution of various pathologies such as coronary failure, infectious, and autoimmune diseases, among others [12,13]. Fibrillar collagen is the main component accumulated during the cardiac fibrosis process. During this new histopathological scenario, EMF leads to wall hardening and myocardial rigidity, limiting both contraction and relaxation. In addition, it can cause electromechanical decoupling due to alterations in the interrelation between cardiomyocytes and the ECM support proteins [14].

EMF has two different presentations reparative or reactive. Reparative EMF is related to the structural preservation of the myocardium, which results in scar formation due to tissue damage that is accompanied by myocytes death [15]. Reactive EMF is not directly associated with cell loss and occurs during i) interstitial fibrosis, where the fibrillar collagen expands in the interstitium between cells [16], or ii) perivascular fibrosis, where the collagen accumulates within the adventitia of intramyocardial arteries and arterioles [14,17,18]. Histologically, interstitial reactive fibrosis can be classified on the basis of tissue architecture into compact, irregular, or diffuse patterns [3,19]. The compact type of fibrosis comprises dense areas of fibrotic tissue formed by collagen devoid of myocytes, whereas the irregular type of fibrosis is characterized by areas wherein collagen fibers in termingle into long filaments and myocardial bundles, inducing branches in the geometry of the tissue.

Researchers have studied cardiac fibrosis using *in silico* platforms, modeling the conditions of the pathology and including cellular interactions and electrical behavior [20–22]. However, it is necessary to conduct *in vitro* validation studies that emulate the physiological conditions of myocardial fibrosis and the spatial distribution of patterns derived from these interactions in controlled microenvironments.

To this end, in the present study, the cellular interactions of cocultures in monolayers of 3T3 fibroblasts and RL-14 cardiomyocytes were determined via the development of a platform of specific cellular patterns using a cell patterning technique; this structure emulated the biomimetics of compact and irregular EMF patterns.

## Materials and methods

### Materials

For the maintenance of cell cultures, Dulbecco’s modified Eagle’s medium (DMEM; 4.5 g/L glucose with L-Gln; Lonza), stabilized penicillin–streptomycin solution (Sigma- Aldrich), and fetal bovine serum (FBS) premium (Biowest) were used. In addition, for the preparation of the substrate for the cell patterns using Poly-L-lysine solution (Sigma-Aldrich), Tergazyme® enzyme detergent (Sigma–Aldrich), and absolute ethyl alcohol (Merck) were used. Finally, for inmunofluorescense Triton X-100 (PancReac Applichem), PBS (Lonza), formaldehyde (PancReac Applichem), Bovine Serum Albumin (BSA) Fraction V (Merck), antivimentin monoclonal antibody (Alexa Fluor® 555) ab203428 (ABCAM), Hoechst 33258 (Sigma-Aldrich) were used.

### Methods

This investigation declared the absence of experiments involving humans or animals. The Health Research Ethics Committee of the Universidad Pontificia Bolivariana approved the study.

#### *In vitro* model: 3T3 fibroblasts and RL-14 cardiomyocytes

For the *in vitro* tissue model cell cultures from the human fetal ventricular cardiomyocyte line RL-14 (ATCC® PTA-1499TM), transformed by the monoclonal antibody SV-40 [23], and 3T3 fibroblasts derived from embryonic mouse cells obtained from a continuous protocol 3T3 cell transfer were used [24]. RL-14 cardiomyocytes have been used in cocultures with other cell lines, due to their similarity in structural and morphological characteristics with native myocytes [25,26]. Likewise, 3T3 fibroblasts have been used in the development of *in vitro* models in the cardiovascular area, including studies for the evaluation of behaviors associated with fibrosis [27,28].

Cultures of human fetal ventricular RL-14 cardiomyocytes and 3T3 fibroblasts were maintained at 37°C in DMEM media with 10% (vol/vol) FBS and supplemented with penicillin (100 U/mL) and streptomycin (100 μg/mL) under conditions of 5% CO_2_, 95% O_2_, and 95% relative humidity [29]. Once the cells had reached a confluence of ≥ 80%, the culture medium was changed, and every 25 passes, the cells were discarded [26].

#### Coculture of fibroblasts and cardiomyocytes

We set up cocultures with RL-14 cardiomyocytes and 3T3 fibroblasts in a monolayer allowing for cell–cell direct interactions, following the technique reported by Rother et al.[30]. Additionally, we used independent control cultures for each cell line. The coculture was maintained in a nutrient medium (DMEM) and supplemented with FBS and antibiotic; it was incubated at 37°C in an atmosphere with 5% CO_2_ and 95% O_2_ and a relative humidity of approximately 95%. The culture medium was changed every 48 h.

Were used patterning techniques to emulate the histopathological patterns (geometric distribution) in the cardiac tissue caused by EMF.

#### Design of cellular patterns with specific geometries

The geometric design of the patterns emulating the EMF tissue distribution was based on images of explanted cardiac tissues with different types of fibrosis, as reported by [6].

For this investigation, was designed a total experimental area of 3.23 cm^2^ for the irregular and compact patterns; these areas were deemed sufficient to evaluate cell–cell interactions, which were compared with tissue microarchitectures from pathological studies in the literature.

**Fig 1:**
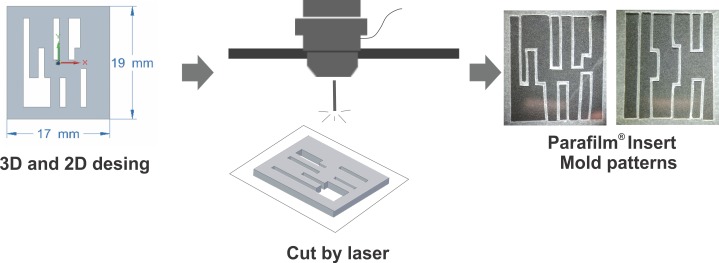
Schematic representation of the generation of mold structures for Parafilm^®^ inserts, emulating the microarchitecture of irregular and compact EMF.

Were used the Solid Edge^®^ and Corel Draw^®^ software to model the structures of the irregular and compact endomyocardial fibrosis in 3D and for the 2D design of the specific cellular pattern architectures. Subsequently, the mold patterns were printed in 3D in acrylonitrile butadiene styrene (ABS) and were cut by a laser on a GS plus 9060 machine in acrylic sheets. Finally, molds were generated in Parafilm®, which emulated the geometric distributions of the EMF. Finally, molds in Parafilm® were generated, which emulated the geometric distributions of the EMF (**Fig 1**).

#### Cell patterning: Specific cellular patterns

Were used an Optika stereomicroscope to visualize the structures compact and irregular on Parafilm® and permit the cuts of knife relevant experimental assay areas using 0.01 mm dissection needles and a No. 11 surgical. Then, we subjected the patterns from the Parafilm® with specific microarchitectures to a protocol of disinfection and sterilization in 1% tergazyme solution and 70% cold ethanol, with successive washes with distilled water. Finally, they were exposed to ultraviolet light before interaction with the *in vitro* model of 3T3 fibroblasts and RL-14 cardiomyocytes.

**Fig 2:**
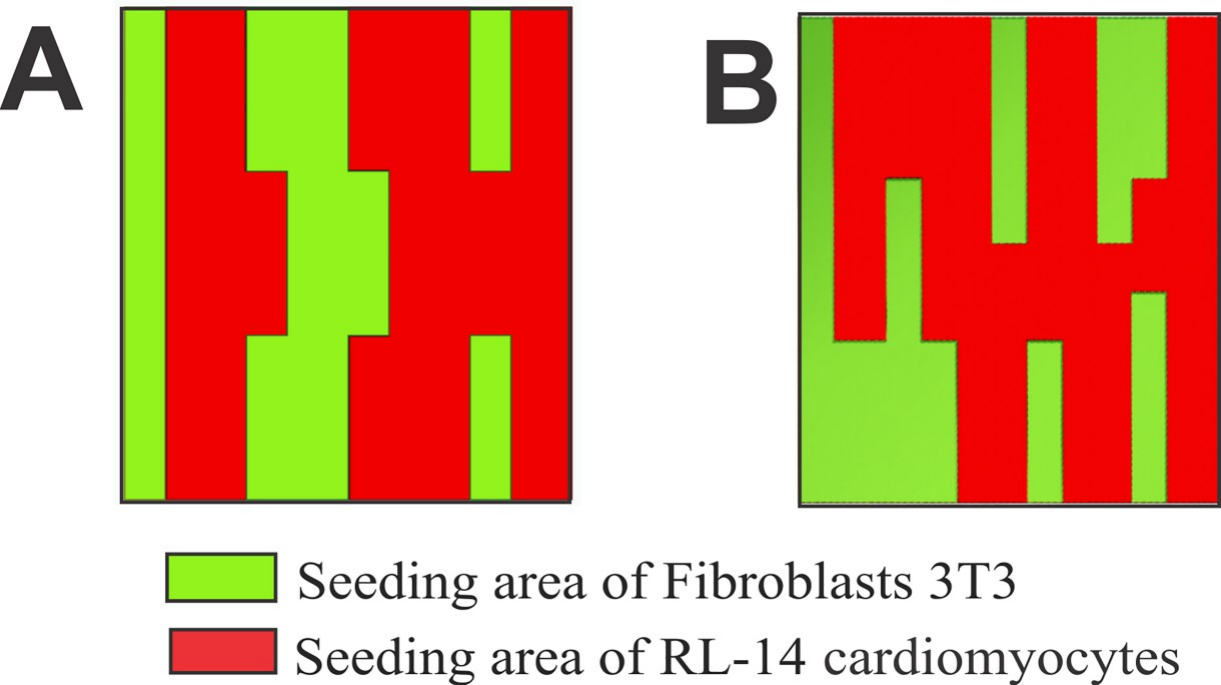
3D structure of the pattern. (A) Pattern emulating a compact EMF. (B) Pattern emulating an irregular EMF.

Was modified techniques reported by Javaherian et al. and Paz [31,32] and used Parafilm® inserts, replicating the patterned characteristics of EMF with irregular and compact spatial distributions. In addition, the microstructure area cell densities for each cell line using a linear control pattern interaction were determined. To do this, we arranged the cell patterns developed in Parafilm® onto petri dishes. To guarantee the coexistence of the cell lines, the cell patterning technique allowed to replicate the behavior of EMF in compact and irregular patterns. **Fig 2** shows 3D models of compact and irregular patterns, with spatial distributions of the experimental zones for each cell line highlighted. Light green color denotes 3T3 fibroblasts, and red color denotes interactions with RL-14 cardiomyocytes.

To ensure adequate cell adhesion, the substrate surface was pretreated with 0.01% Poly-L-lysine before placing a population of each type of cell line (fibroblasts or cardiomyocytes) on the work areas. After 24 h, the Parafilm® sections limiting the interaction areas between both cell lines were removed and changed the culture medium. Finally, cell adhesion and proliferation was evaluated after incubation periods of 24, 48, and 72 h using light-field microscopy.

#### Evaluation of the cellular interactions and morphologies

The behavior of the spatial distribution, adhesion, and selective cell proliferation was noted by light-field microscopy and recorded by observations with an Optikam Pro 5 camera. Likewise, hematoxylin–eosin (HE) staining was used to visualize cell morphology changes of the fibroblasts and cardiomyocytes interacting in the pattern of each defined microarchitecture. For the analysis of the cellular patterns, a 4× magnification was used to provide large visual fields of the study area.

#### Immunofluorescence staining

For immunofluorescence the cocultures were fixed with 4% formaldehyde for 15 minutes at room temperature, then washed with 1X phosphate buffered saline (PBS), then the Parafilm^®^ mold structures were removed, permeabilized with 0.1% Triton X-100, blocked with 1% bovine serum albumin (BSA) in PBS for 1 hour and incubated with the anti-vimentin monoclonal antibody conjugated with Alexa Fluor 555 (1:1000) diluted in PBS containing 0.1% BSA. Subsequently nuclei were stained with Hoechst 33258.

#### Flow cytometry

To perform a population cell count of fibroblasts and cardiomyocytes, carboxyfluorescein succinimidyl ester (CFSE) fluorophore diacetate was used, which is a membrane marker for cell proliferation studies, at a concentration of 5 μM, detected in a BD LSR Fortessa^^™^^ cytometer at a wavelength of 488 nm. Labeling with CFSE allowed to quantify the cell growth and proliferation, and indirectly the interaction of the two cell lines present in the patterns that emulate EMF.

To identify the populations of each cell line present in the patterns, the RL 14 cardiomyocytes were labeled with CFSE in order to generate a positive label for CFSE and a negative label for 3T3 fibroblasts.

#### Image processing

The interactions of cardiomyocytes and fibroblasts using the Bio-EdIP software with images obtained by light-field after HE staining were quantified. This software allowed quantifying the percentages of cell confluence. In addition, the size of the 3T3 fibroblast cell nucleus and the RL-14 cardiomyocyte cocultures of the images registered by fluorescence microscopy was determined using the Fiji^®^ software. Finally, for the quantification of the fluorescence intensity of the vimentin expressed in fibroblasts, which plays an important role in the evolution of fibrosis, the measurement of the profiles of the red channel corresponding to the emission of alexa fluor 555 was performed. The mean intensity values obtained from the individual immunofluorescence images were used for the statistical analysis. Nine images were processed and analyzed for each type of irregular and compact pattern and coculture control; in total 27 samples were obtained from a 2^3^ experimental design.

#### Statistical analysis

Multifactorial ANOVA analysis was performed, which allowed to determine the standard deviations, population means, multiple range tests, minimum significant differences (Fisher’s LSD test), and statistical significance with P values of < 0.05. In addition, the 27 study samples were considered in triplicates in three independent experiments for each of the following three parameters: selective adhesion, spatial distribution, and cell morphology. The open area and cellular areas were considered as dependent variables, and the incubation period and type of pattern were considered as factors that influenced the model.

In the present study, we defined the cell cultures with specific cell distributions for each type of pattern that emulated EMF as cases. Likewise, we defined the cell cultures without any patterns as controls.

## Results and discussion

### Coculture development

**Fig 3:**
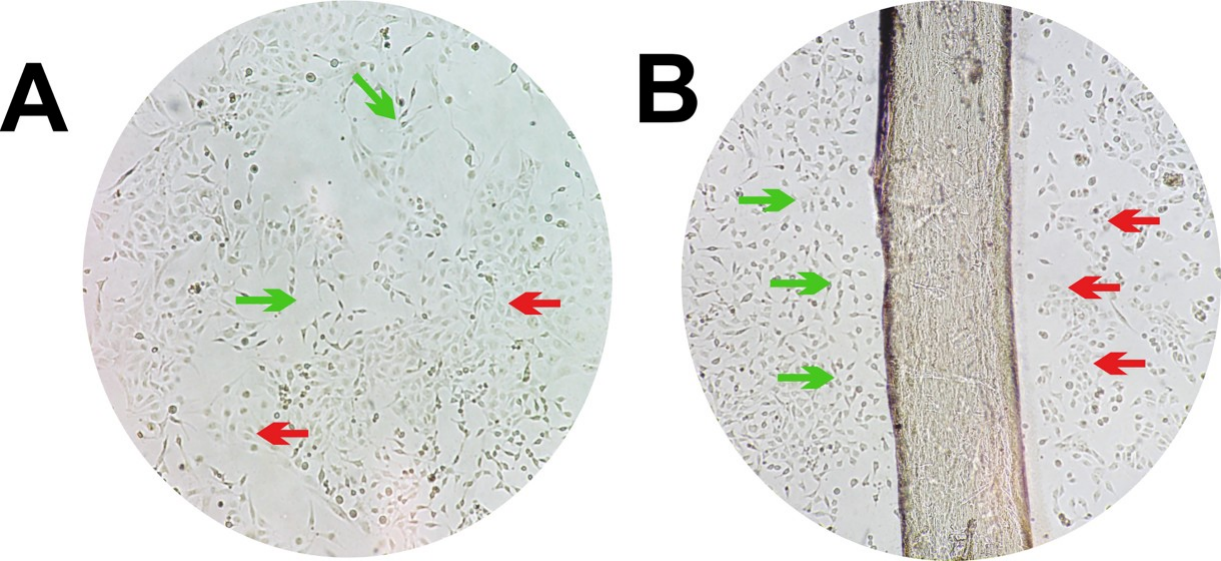
Coculture development. 10× magnification. (A) Control coculture of RL-14 cardiomyocytes and 3T3 fibroblasts. The red arrows point to cardiomyocytes and the light green arrows to fibroblasts. (B) Culture of RL-14 cardiomyocytes and 3T3 fibroblasts arranged in a linear control pattern. The red arrows point to cardiomyocytes and the light green arrows to fibroblasts.

**Fig 3A** shows a coculture control, without any type of predefined geometry, between RL-14 cardiomyocytes and 3T3 fibroblasts, with an approximate cell confluence of 85%. The figure shows the coexistence of the two cell types with a heterogeneous cell proliferation distribution.

A linear control pattern with an area of 15 mm^2^ was used as a basis to determine the initial work conditions of the coculture. Then, mold patterns that replicated the architecture of compact and irregular EMF were developed. In the initial trials, a population density of 2.5 × 10^3^ cells/cm^2^ of RL-14 cardiomyocytes and 3.5 × 10^3^ cells/cm^2^ of 3T3 fibroblasts was sown. From which, it was observed that the resulting cellular density was not sufficient to generate cellular microdomains to enable cellular interactions (**Fig 3B**).

### Initial cell population density analysis

**Fig 4:**
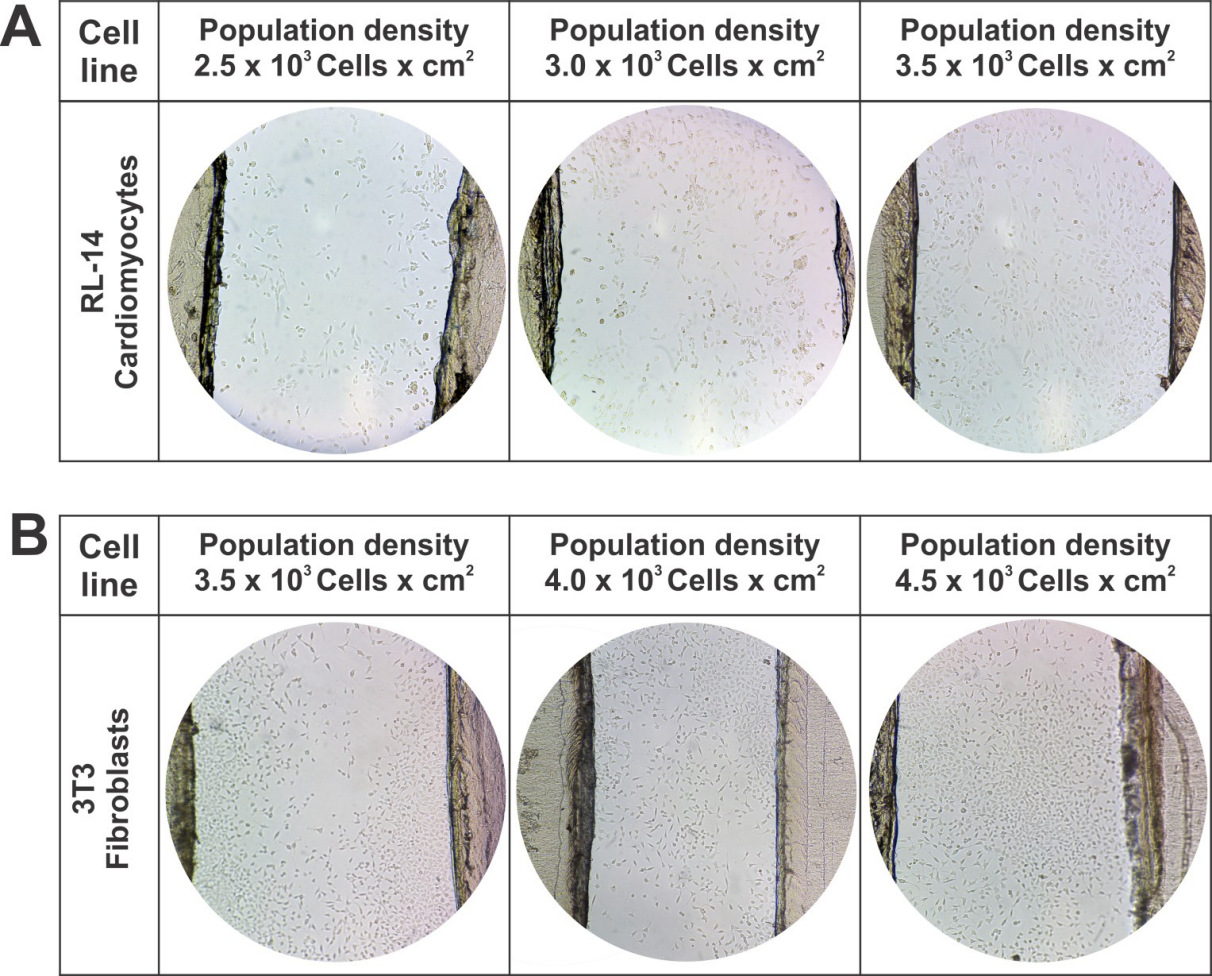
Effect of population density on the linear pattern. 10× magnification. (A) Linear pattern of interaction with RL-14 cardiomyocytes. (B) Linear pattern of interaction with 3T3 fibroblasts.

Due to cell size differences between fibroblasts and cardiomyocytes, it was determined that the cell proliferation disposition in the linear control pattern was affected in relation to the experimental area of the pattern. This behavior is demonstrated in **Fig 4**, wherein three population densities were evaluated for RL-14 cardiomyocytes (2.5 × 10^3^, 3 × 10^3^, and 3.5 × 10^3^ cells per cm^2^) (**Fig 4A**) and three densities for 3T3 fibroblasts (3.5 × 10^3^, 4 × 10^3^, and 4.5 × 10^3^ cells per cm^2^) (**Fig 4B**).

Based on the preliminary results of the linear control pattern, it was determined to choose in the irregular and compact microarchitecture patterns population densities per work area of 3.5 × 10^3^ cells/cm^2^ for RL-14 cardiomyocytes and 4.5 × 10^3^ cells/cm^2^ for 3T3 fibroblasts, confirming that these cellular densities showed good spatial arrangement in the patterned areas.

### Implementation of the cell patterning technique in an *in vitro* model with fibroblasts and cardiomyocyte cocultures

**Fig 5:**
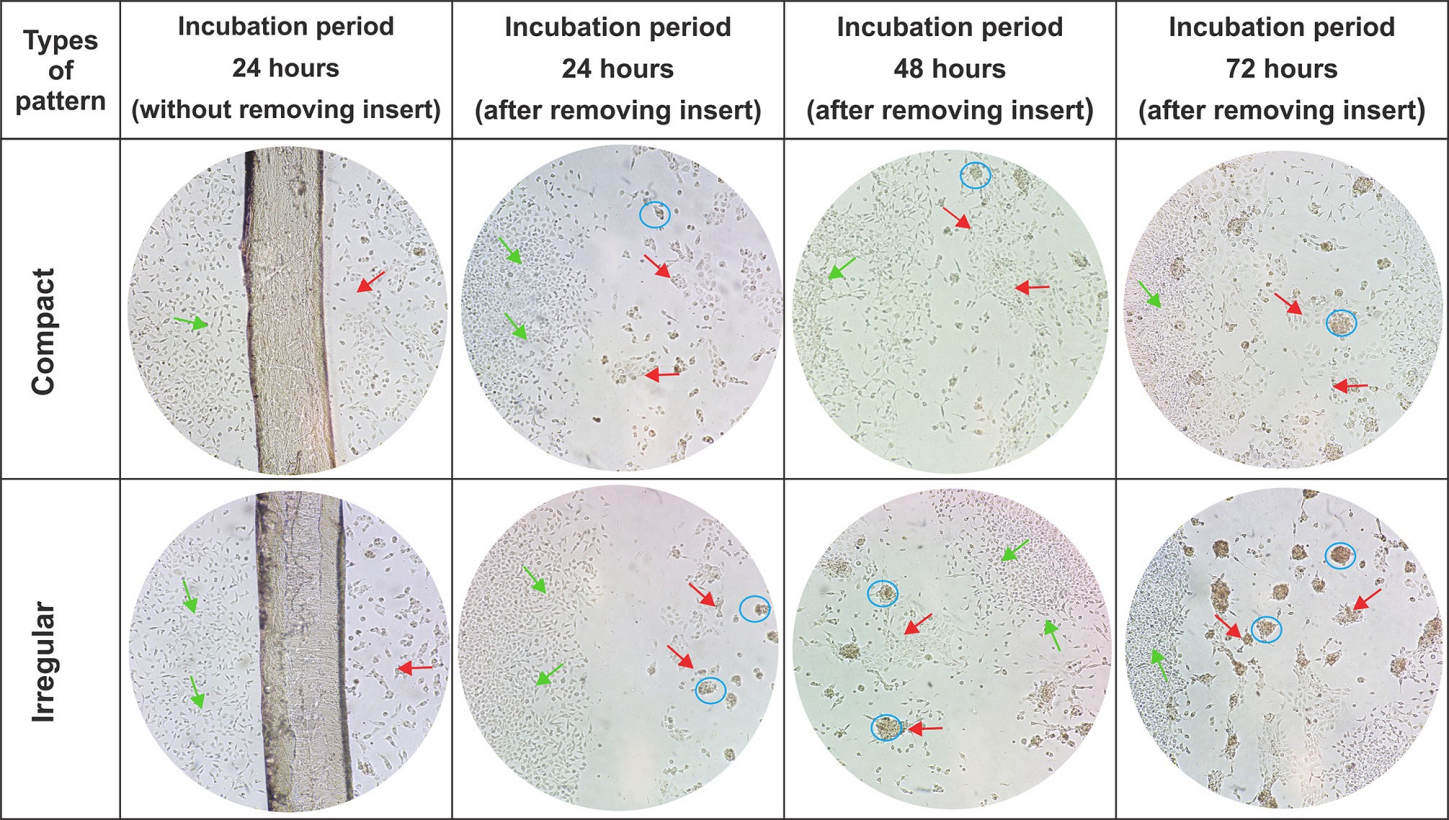
Coculture of RL-14 cardiomyocytes and 3T3 fibroblasts emulating a pattern of compact and irregular fibrosis. Incubation periods of 24, 48, and 72 h. The light green arrows indicate 3T3 fibroblasts; the red arrows indicate RL-14 cardiomyocytes, and the blue circles indicate the presence of cells agglomerations (10× magnification).

EMF of the compact and irregular type differs in the spatial distribution of fibroblasts in cardiac scar tissues. In the present study, it was found that the use of complex geometrically structures could emulate the biomimetics of EMF. In the coculture, the Parafilm® inserts separating each cell line were removed after 24 hours of incubation. This procedure allowed to verify that in both types of cell patterns, adhesion and cell proliferation are favored from the first 24 h of registration. After removal of the Parafilm® inserts, cell agglomerations of the RL-14 cardiomyocytes were observed; this was more noticeable in the irregular patterns than in the compact ones. This effect persisted and increased as the incubation period progressed (**Fig 5**).

The generation of the cellular agglomerates presented in the patterns with irregular and compact distributions **(Fig 5),** is associated with the physical confinement to which the cells were subjected in the defined areas that emulated the EMF. Likewise, the delimited cell growth areas for each type of pattern were reduced as the incubation period increased, due to the increase in the fibroblast cell population. This behavior is similar to that reported in the investigations of Shivashankar et.al and Ankar et.al [33,34], in which cell agglomerates as a result of confined geometries were denoted, which finally led to perturbations in the extracellular matrix topography, an important factor in cell homeostasis.

### Selective cell adhesion, spatial distribution, and cell morphology

**Fig 6:**
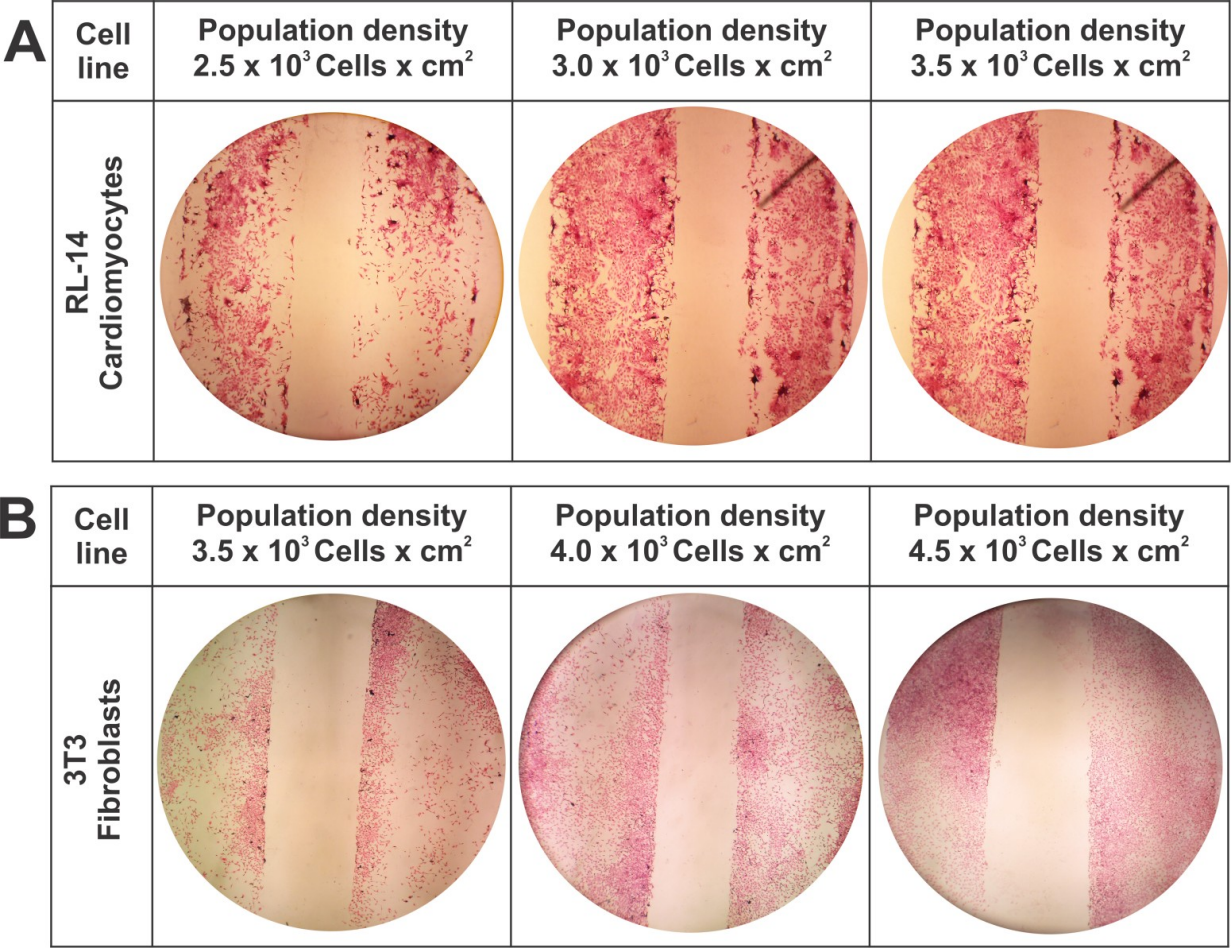
Linear pattern with different cell densities of RL-14 cardiomyocytes and 3T3 fibroblasts by HE staining. 4× magnification. (A) Linear pattern of interaction with RL-14 cardiomyocytes. (B) Linear pattern of interaction with 3T3 fibroblasts.

The cell cultures shown in **Fig 6** highlight the increases in the proliferation rates of cardiomyocytes and fibroblasts as well as the conformation of morphologically viable cell syncytia. It was found that these cell types initially adhere selectively to the linear areas in the control patterns, from these results, population densities for the irregular and compact patterns were emulated.

Regarding the spatial distribution of cell adhesion in the microstructures, we obtained the best response using population densities of 3.5 × 10^3^ cells per cm^2^ for RL-14 cardiomyocytes and 4.5 × 10^3^ cells per cm^2^ for 3T3 fibroblasts. Using these cell densities, all 15 cm^2^ areas per band of the pattern were filled.

**Fig 7:**
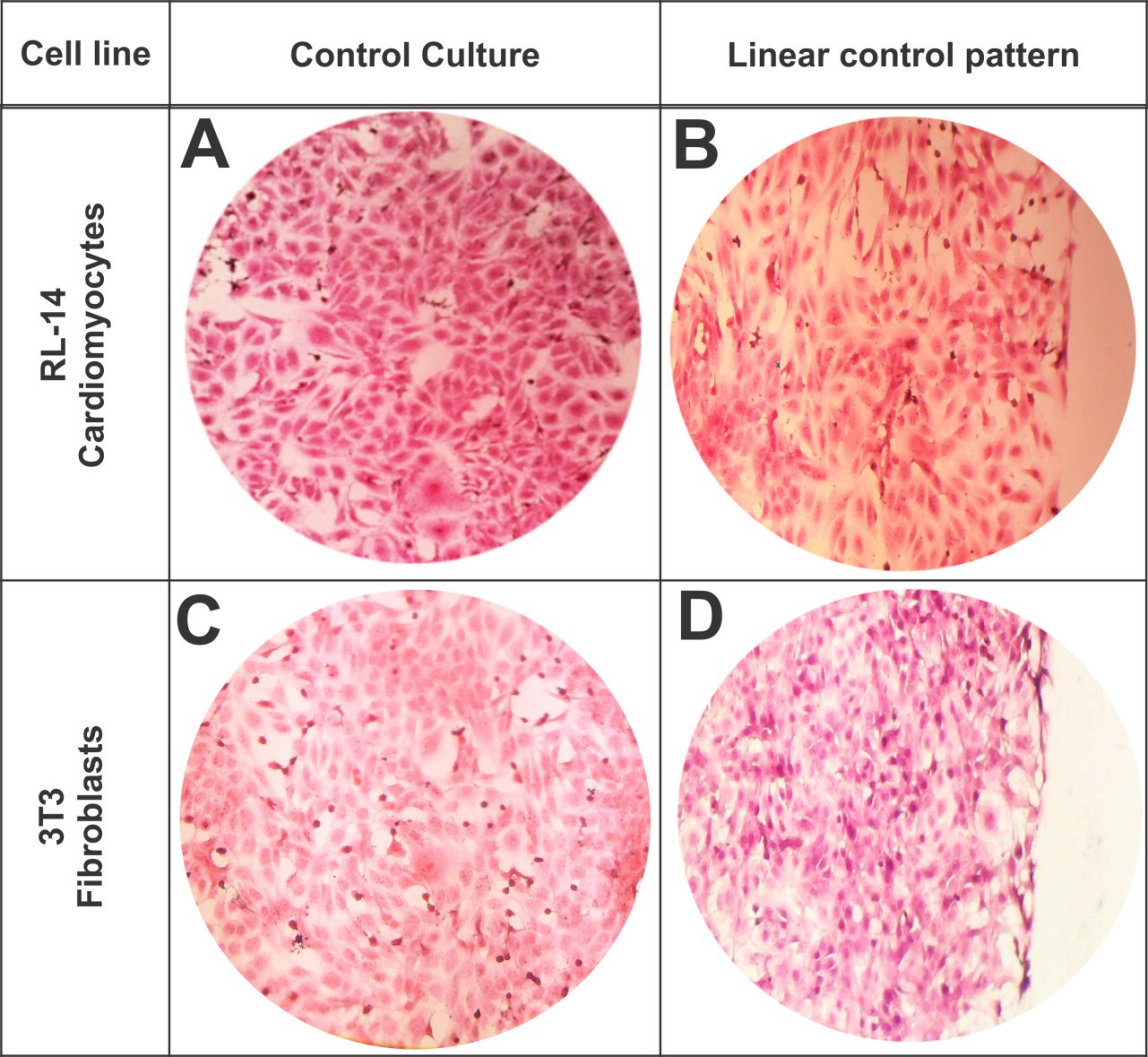
Effect of cell morphology of control cultures without a pattern compared with the linear control pattern. (A) Control culture of RL-14 cardiomyocytes. (B) Linear control pattern culture of RL-14 cardiomyocytes. (C) Control culture of 3T3 fibroblasts. (D) Linear control culture of 3T3 fibroblasts. HE staining. (20× magnification).

It was noted that in the linear control patterns, cell morphology conformations were not affected compared with those in their respective cell control (**Fig 7**). HE staining allowed for visualization of the cellular syncytia of RL-14 cardiomyocytes and 3T3 fibroblasts, likewise, qualitatively assess cell type phenotypes in a linear spatial distribution without border restrictions.

**Fig 8:**
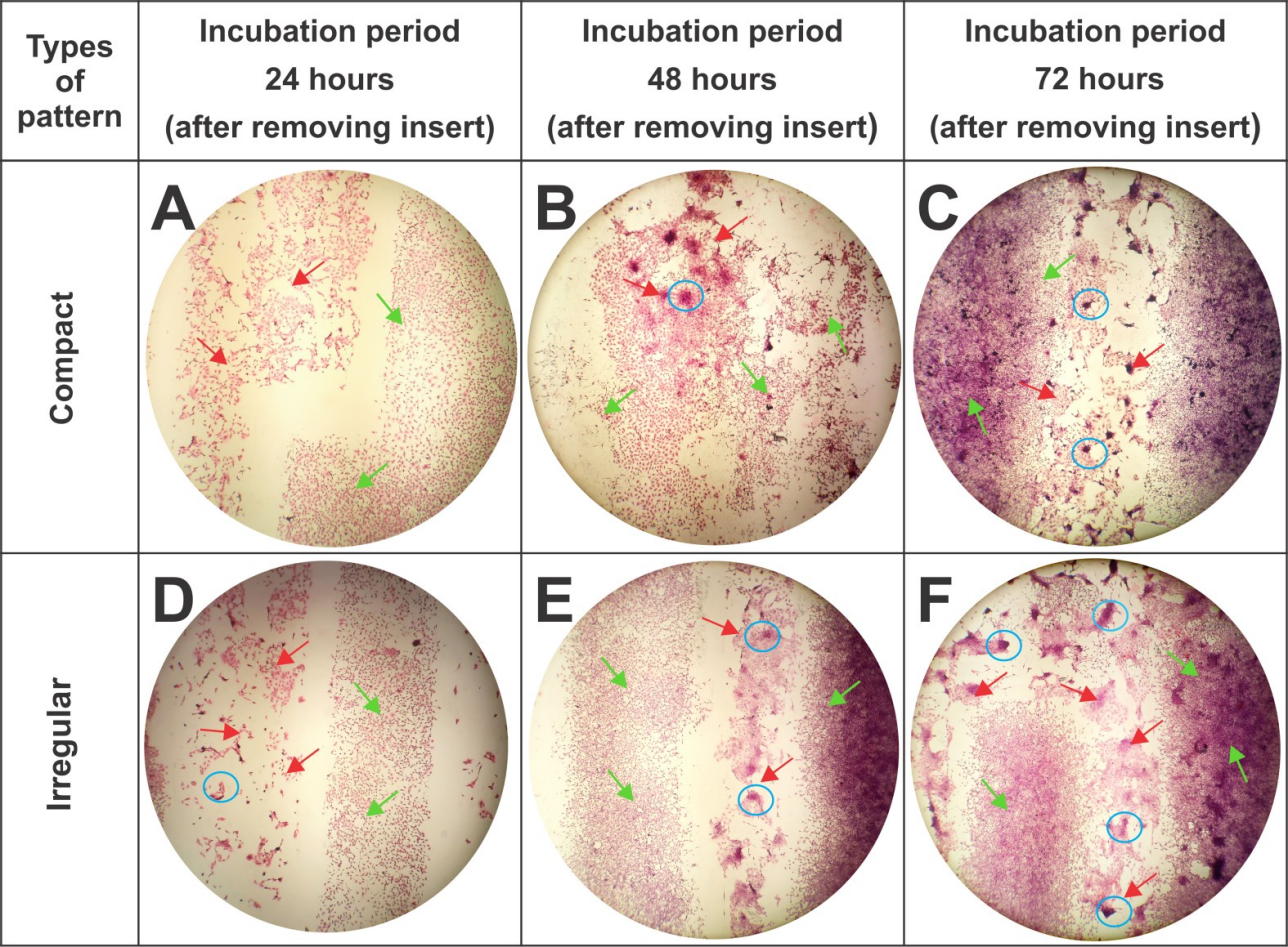
Cellular microarchitectures of compact and irregular patterns incubated for 24, 48, and 72 h. The light green arrows indicate 3T3 fibroblasts; The red arrows indicate RL-14 cardiomyocytes, and the blue circles indicate the presence of agglomerations. (4× magnification). (A) Compact pattern stained after 24 h of incubation. (B) Compact pattern stained after 48 h of incubation. (C) Compact pattern stained after 72 h of incubation. (D) Irregular pattern stained after 24 h of incubation. (E) Irregular pattern stained after 48 h of incubation and (F) Irregular pattern stained after 72 h of incubation. Stained with HE.

**Fig 8** shows the compact and irregular patterns evaluated during incubation periods of 24, 48, and 72 h, with HE stain, which display changes in the morphological characteristics of RL-14 cardiomyocytes and 3T3 fibroblasts. In the compact pattern, during the first 24 h of incubation and after removing the Parafilm^®^ separation bands of Parafilm^®^ (**Fig 8A**), an increase in cellular proliferation rates, selective adhesion, and conservation of both cell line morphologies compared with the same variables in control cultures was observed. In addition, after 48 h of incubation (**Fig 8B**), the cell proliferation rate increased, facilitating the connectivity between both cell lines: but agglomeration of RL-14 cardiomyocytes was noted in some areas. At the end of 72 h of incubation, an increase in the size of cardiomyocyte agglomerates was observed, which could indicate cell loss in areas corresponding to these cellular structures.

Likewise, **Fig 8** shows the morphological characteristics of the irregular patterns. After 24 h of incubation and after removing the Parafilm insert (**Fig 8D**), an increase in cell proliferation of 3T3 fibroblasts compared with RL-14 cardiomyocytes was observed, in the latter, the RL-14 cardiomyocytes showed some agglomeration. After an incubation period of 48 h (**Fig 8E**), it became clear that as the population density of cardiomyocytes increased, the formation of agglomerations also increased. Finally, after 72 h of incubation (**Fig 8F**), more connectivity between both cell lines was observed, but the presence of agglomerations in the area of RL-14 cardiomyocytes also increased, which resulted in the loss of its morphological characteristics compared with those in the control. The morphological changes of cardiomyocytes did not affect the cellular morphology of 3T3 fibroblasts, which was also compared with that in their control pattern.

Mosadegh and colleagues [35], report morphological changes in cardiomyocytes in presence of fibroblasts, going from an elongated to a rounded shape and a decrease in the cell density of cardiomyocytes, likewise, Moreno et al. [36] found alterations in the cell membrane and possible changes in mitochondria of cardiomyocytes undergoing processes such as hypoxia and myocardial ischemia; these behaviors are similar to obtained in the present study in the microstructures that emulated the EMF of compact and irregular type, evidencing loss of in the cellular density and alterations in the form of the cardiomyocytes.

**Fig 9:**
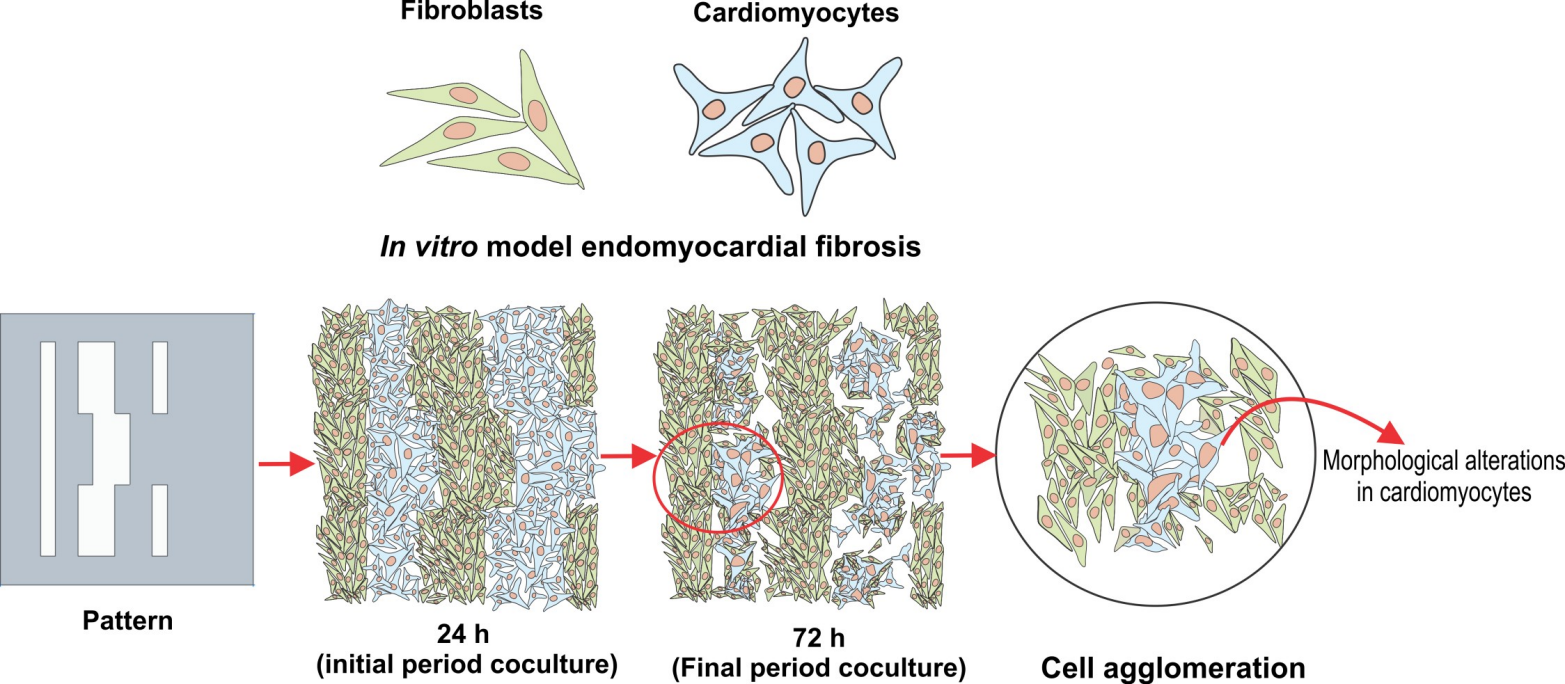
Interaction scheme of patterning of fibroblasts and cardiomyocytes emulating EMF.

**Fig 9** shows a representative scheme of the cell distribution that both cell lines adopted during the cell patterning process that emulated the EMF microarchitecture. In this context, in endomyocardial fibrosis it is assumed that the loss of a significant number of cardiomyocytes can trigger reparative processes that lead to the formation of fibrotic tissue, likewise, the death of myocytes can generate the release of cytokines and chemokines triggering inflammatory responses as a result of chemotactic processes. Analyzing the results obtained, it was found that this behavior coincides with those presented by Zhao et al [37], which described that in cardiac fibrosis there is an absence or significant loss of cardiomyocytes, due to pressure or volume overload, brief or repetitive ischemia, and deterioration of cell morphology.

### Cellular area analysis

**Fig 10:**
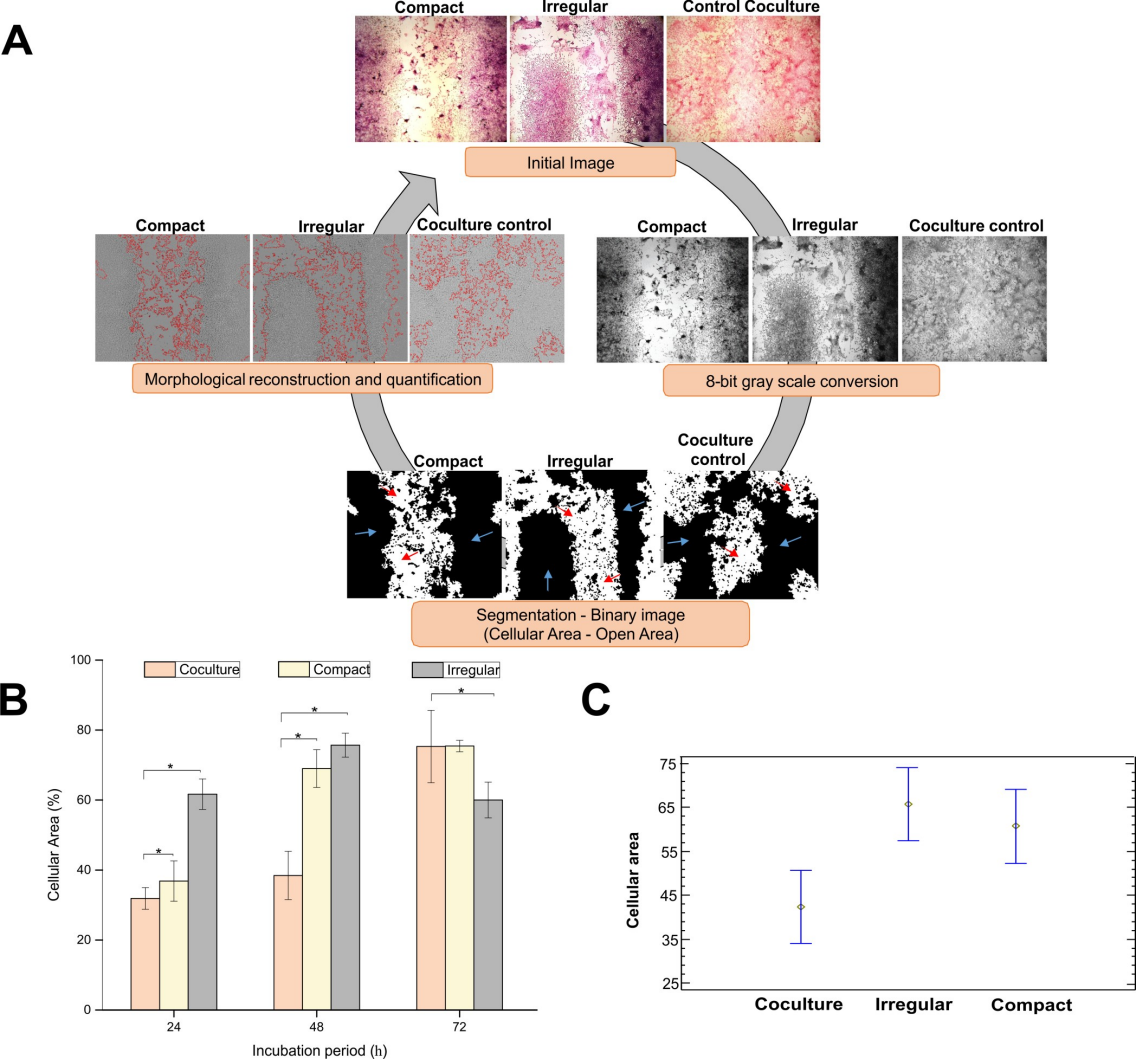
Quantification of cellular and open areas. (A) Image processing scheme in the Bio-EdIP software for the cellular and open areas. The red arrows indicate the open area (**S1 Fig**), and the blue arrows indicate the cellular area. (B) cellular area populated in the compact and irregular patterns for incubation periods of 24, 48, and 72 h where * p < 0.05 in relation with the coculture control (C) Populations means of the cellular area in relation to the pattern type. The data are presented as mean ± standard error (n = 3).

The Bio-EdIP software allowed to images processing of compact and irregular patterns obtained by light-field microscopy and the coculture control stained with HE. **Fig 10A** shows the quantification process of the images by means of segmentation and morphological reconstruction.

The values corresponding to the cellular areas for the incubation periods of 24, 48, and 72 h for each type of pattern compared with the areas in coculture controls, and the cell proliferations increased with extended incubation periods (**Fig 10B**). After 24 h, the cellular areas occupied by the populations of cardiomyocytes and fibroblasts seeded for each type of pattern were 36.84% for the compact pattern, 61.67% for the irregular pattern, and 31.57% for the coculture control. After 48 h of incubation, the cellular areas increased by 32.16% in the compact pattern, 14% in the irregular pattern, and 6.57% in the coculture. However, at the end of the incubation period of 72 h, a decrease of 15.6% in the cellular area of the irregular pattern was observed, which differed from our finding for the compact pattern, which had increased by 6.44%. Meanwhile, the coculture area had increased by 36.86%. These results are related to the pattern microarchitectures, which result in different cell densities associated with the geometric distribution of cardiomyocytes and fibroblasts interacting.

Likewise, the results showed that after 24 h of incubation, the cell population had increased by 4.96% on the compact pattern and by 29.79% on the irregular pattern compared with the population in the coculture control. The same trend was conserved after 48 h of incubation, wherein the compact pattern increased its cell population by 30.55% and the irregular pattern by 37.23% in relation to the coculture population. However, after 72 h of incubation, the cell density of the irregular pattern markedly decreased by 15.29% compared with that in the control, but the compact pattern increased only by 0.14% in relation to the coculture control. The open area for each of the patterns compared with the area in the coculture control, and the trend is the opposite of that presented in the graph of cellular areas (**Fig 10B**). This difference is due to the increase in cell proliferation with time, reflecting the decrease in the percentage of open area. These results could be due to the loss of myocytes and the increase in cellular agglomerations as a consequence of chemotaxis [35] in the presence of 3T3 fibroblasts.

The results obtained in the present work showed patterns that replicated the EMF of compact and irregular type, in which areas with presence of agglomerations in the zones corresponding to the cardiomyocytes were observed, this behavior could be adduced to the metabolic stress presented in the cardiomyocytes, which produce cytokines that could induce an increase in the proliferation of the fibroblasts and migration to injured areas (chemotaxis) due to the increase in the concentrations of cytosines. These results are consistent with those obtained by Mosadegh et al. and Bujak et al. [35,38], which indicate that the signaling molecules expressed by cardiomyocytes are chemoattractants, likewise, Sadeghi and colleagues [39], found the presence of factors involved in the chemotaxis processes, such as transforming growth factor β1 (TGF-β1), which plays an important role in the development of tissue fibrosis, and when increased induces hypertrophy in cardiomyocytes causing chemotaxis and elevated MEC secretion by fibroblasts. Alike, these studies report mechanobiological factors such as tissue stiffness, mechanical stress and hemodynamic stress.

An ANOVA statistical analysis in this study was used for a reproducible platform to emulate the biological characteristics of the compact and irregular EMF microarchitectures. This analysis allowed to determine the mean populations of each one of the replicas in the three incubation periods evaluated. **Table 1** show the increase or decrease in cell densities affected by specific pattern distributions. The range of the statistical significance of the variable modifying factors was determined, which indicated that the cellular areas of the irregular and compact patterns were statistically significantly different at 23.51% and 18.41%, respectively, compared with those in the coculture controls. This is contrary to what happens when comparing the cellular proliferations of the irregular and compact patterns, wherein an increase in the cell population was observed without a statistically significant difference because both patterns had a similar increase in cell density (**Fig 10C**). These graphics indicates cellular area changes in relation to the pattern type.

**Table 1:** Percentages of cellular areas in the compact and irregular patterns.

Incubation Period (h)	Cellular Area (%)		
	Coculture Control	Compact P.	Irregular P.
24	31.876 ± 9.2795	36.836 ± 17.254	61.674 ± 13.089
48	38.446 ± 20.678	69.791 ± 16.195	75.674 ± 10.281
72	56.508 ± 31.024	75.440 ±± 4.889	60.018 ± 15.305

*The data are presented as mean ± standard deviation with 95% confidence intervals (n = 3)

### Immunofluorescence

**Fig 11:**
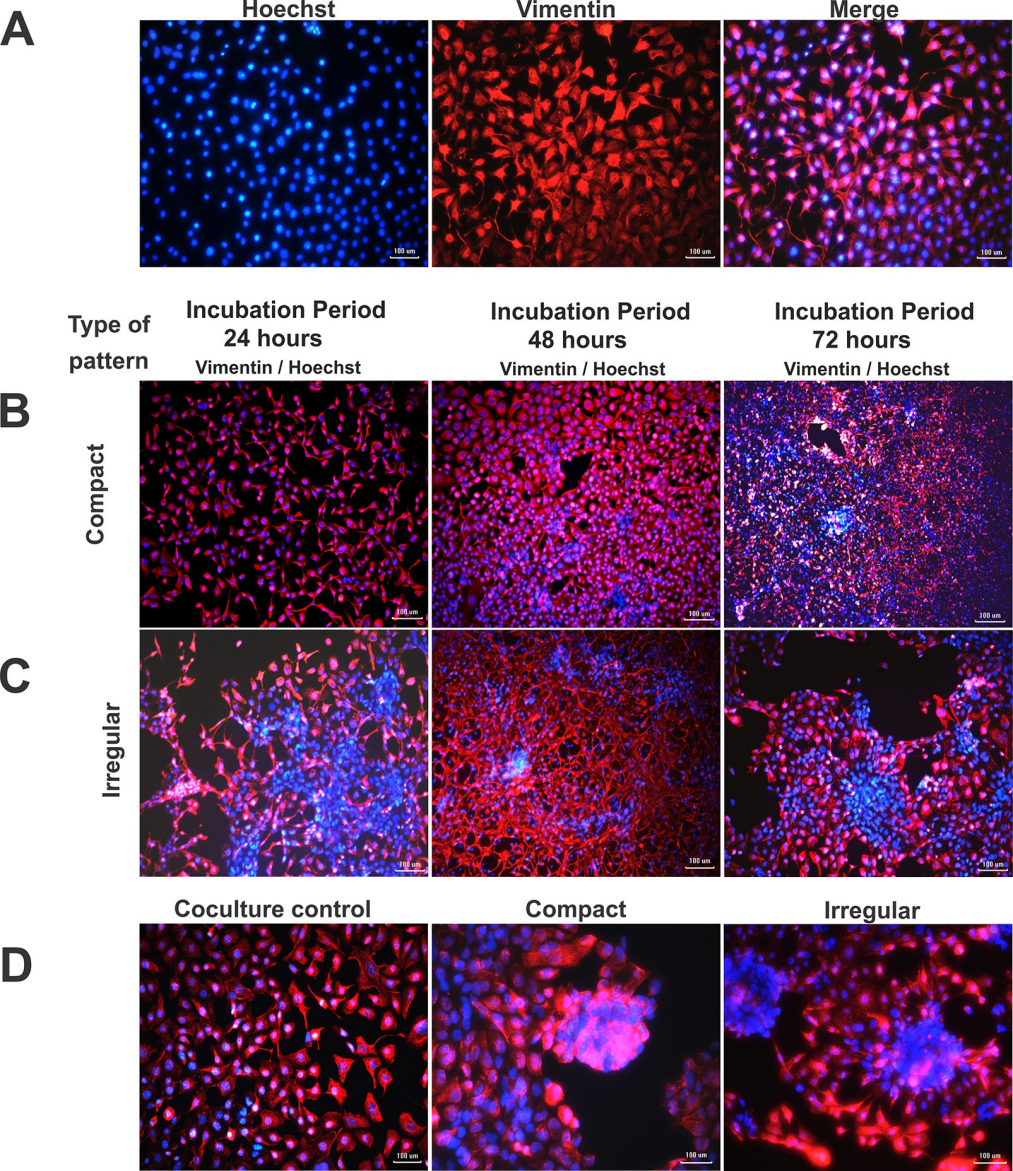
Cell patterns emulating microarquitectures of EMF of compact and irregular type and coculture control pattern. Incubation periods of 24, 48 and 72 h. (A) Coculture control with vimentin, hoechst and merge labeling. (B) Linear compact pattern. (C) Irregular pattern. (D) Evidence of agglomerations or cell fusions (incubation period of 48 h). Cytoskeleton intermediate filaments (vimentin, red) and nuclei (hoechst, blue). The patterns show the merge stain. 10× and 20× magnification.

**Fig 11** shows the cellular patterns emulating the microarquitectures of the compact and irregular type of EMF, in addition, the coculture control pattern is presented. Positive labeling vimentin by means of immunofluorescence was evidenced, which was used for the detection of intermediate cytoskeleton filaments of 3T3 fibroblasts, a cell type that increases its expression in pathological fibrosis processes. Likewise, the nuclei corresponding to RL-14 cardiomyocytes and 3T3 fibroblasts were identified using hoechst (**Fig 11A**).

In the compact pattern, positive labeling for hoechst and vimentin was evidenced (**Fig 11B**), denoting a homogeneous cellular network at a 24 h incubation period, in which it was observed that as the incubation period increased the densities of cardiomyocytes and fibroblasts populations increased. Likewise, during the 48 h incubation period generation agglomeration was denoted (**S2 Fig**), behavior that increased at 72 h of incubation of both cell lines.

On the other hand, in the irregular pattern (**Fig 11C**) the presence of agglomerations was identified from the first 24 h of interaction and as the incubation period increased greater expression of vimentin and generation of connection fibers between the fibroblasts was denoted. Finally, in the 72 h incubation period there was a decrease in the cell density of cardiomyocytes and an increase in agglomerations (**S3 Fig).**

In compact and irregular patterns agglomerations corresponding to cellular structures of the RL-14 cardiomyocytes were observed (**Fig 11D**), alike, the formation of structural networks by 3T3 fibroblasts were denoted, which migrated and invaded zones with presence of cardiomyocytes causing alterations in the nucleus size; these interactions favored the induction of morphological changes, fusion and possible cell death. The behaviors found in this research are similar to those reported by clinical and research studies of the EMF, where in the different microstructural conformations of the affected tissue there is cellular loss of the myocytes and substitution to fibrotic tissue, This is related to an increase in the population of fibroblasts and in turn overexpression in vimentin levels [37,40–42]. On the other hand, the presence of agglomerates in **Fig 11D** could be related to increases in TGF-β expression, which induces the formation of fibrotic nodules composed of cells embedded in ECM. These findings have related to those reported by Xu et.al [43], in which the formation of nodules was dependent on the increase in TGF-β, involving greater cell migration and increasing the production of ECM, which causes alteration in cellular dynamics.

Given these results, it was concluded that the irregular pattern with respect to the compact pattern showed a decrease in the cell density of cardiomyocytes, large agglomerations and loss of cell morphology. These behaviors in the compact and irregular pattern are consistent with those found by Camelliti et al. which determined that the increase in fibroblast proliferation causes migration to areas with tissue lesions and a decrease in myocyte cell density. Likewise, Laframboise and his group [44] reported that excessive proliferation of the fibroblast population in interaction with cardiomyocytes induces structural and functional alterations.

### Size and circularity of cell nuclei

**Fig 12:**
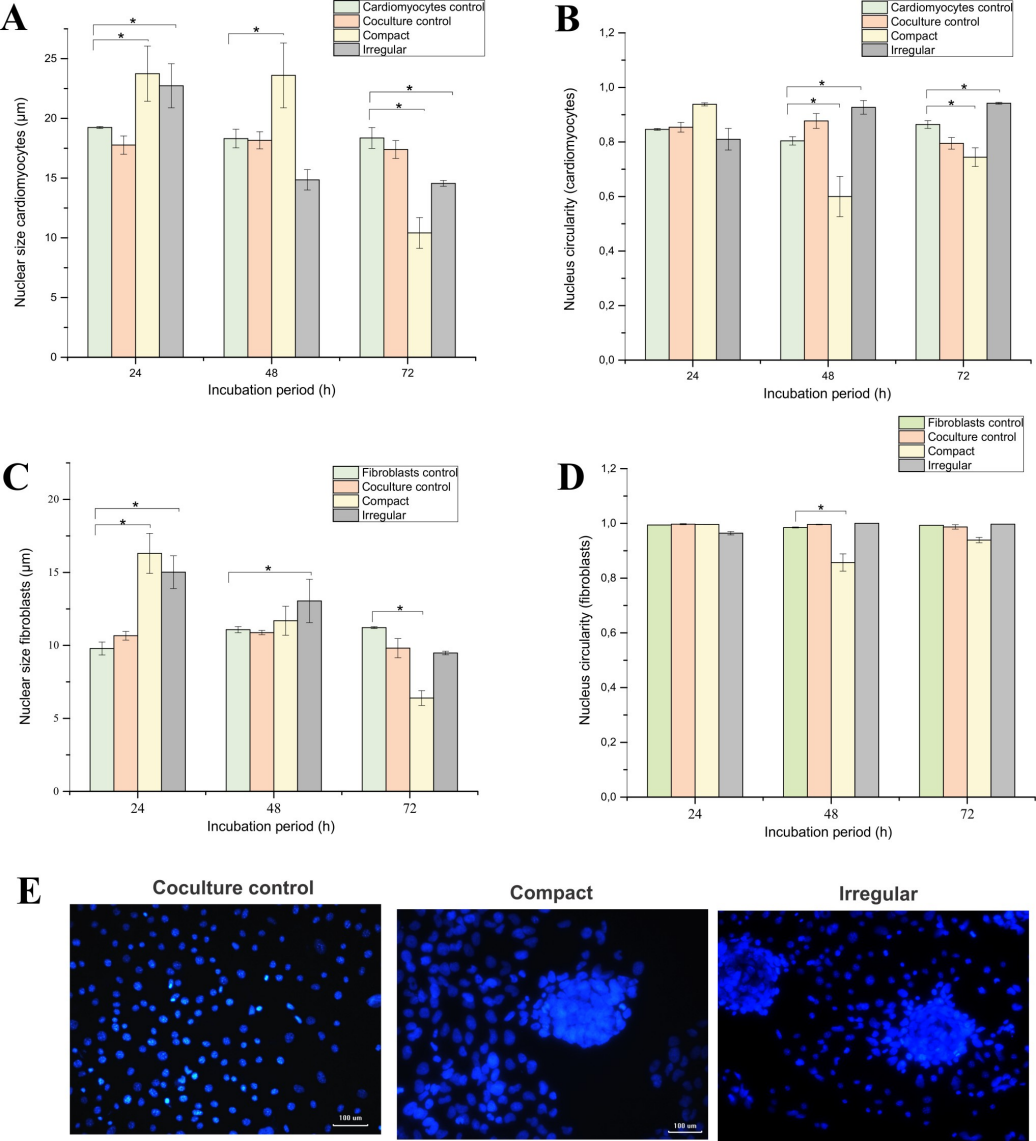
Size and circularity of nuclei of the RL-14 cardiomyocytes and 3T3 fibroblasts in relation to the type of pattern. Incubation periods of 24, 48 and 72 h. (A) nuclei size of RL-14 cardiomyocytes, (B) Circularity of the nuclei in the RL-14 cardiomyocytes, (C) nuclei size of 3T3 fibroblasts. (D) Circularity of the nuclei in the 3T3 fibroblasts. (E) coculture control nucleus labeling, compact and irregular pattern (hoechst, blue and 20× magnification). The data are presented as mean ± standard error (n = 3).

Given the results obtained by immunofluorescence, the Fiji^®^ software was used to determine the sizes and circularity of the cell nuclei of the RL-14 cardiomyocytes and the 3T3 fibroblasts seeded by separately, in coculture and in the patterns that replicated the compact and irregular EMF (**Fig 12**). Therefore, it is important to highlight with **Fig 12** that the study of this type of alterations can lead to changes in the morphology of the cells that affect the intermediate filaments of the cytoskeleton and nuclear expression [33], likewise could alter the biochemical signaling pathways and its subsequent cellular response [45]. This analysis allowed to identify the morphological changes of the nuclei associated with the increase or decrease in size and loss of circularity, as the two cellular models interacted.

The size of the cardiomyocyte cell nucleus without interaction with fibroblasts (cardiomyocyte control) showed an apparently constant behavior without a statistically significant difference, in which the size varied 0.93 μm and 0.88 μm between the incubation period of 24 h versus 48 h and 72 h respectively. Likewise, it is shown that the coculture control presented a behavior similar to cardiomyocyte control. The compact pattern in the incubation period from 24 h to 48 h did not show apparently significant changes in the size of the nucleus, but at the incubation period from 24 h to 72 h it exhibited a decrease of 13.33 μm. Finally, in the irregular pattern there was a decrease in the size of 7.9 μm and 8.2 μm for the incubation periods of 48 h and 72 h respectively, however, among these incubation periods the nuclei size did not have a representative variation (**Fig 12A**).

The nucleus size of the fibroblast controls denoted an increase of 1.3 μm and 1.4 μm for the incubation periods of 24 hours versus 48 and 72 h, respectively, where the size variation was not statistically significant. Likewise, the coculture control did not show statistically significant variation in the incubation period of 24 h versus 48 h, while the size decreased to 0.85 μm at a period of 72 hours. The compact pattern showed a decrease of 4.61 μm and 9.41 μm for the incubation periods of 48 and 72 h. respectively. Finally, the irregular pattern denoted a reduction of 1.97 μm and 5.53 μm as the period of cellular interaction increased (**Fig 12C**).

Given the results obtained, 3T3 fibroblasts do not show alterations in the degree of circularity of their nuclei (**Fig 12D**), assuming that circularity or integrity is preserved at values close to 1 and disorders at values close to 0, while the cardiomyocytes presented variations of the circularity with a tendency to move away from the value of 1 (**Fig 12B**) [46]. It should be verified if this behavior occurs in response to the evaluated microarchitectures that emulated EMF, due to the fusion, activation or chemotaxis of fibroblasts in interaction with cardiomyocytes.

**Fig 12E** shows the representative morphological changes in the nuclei related to each type of coculture control, compact and irregular pattern, which denote changes in the size and circularity associated with the formation of agglomerations.

The changes presented in the nuclei of cardiomyocytes in interaction with fibroblasts in the EMF model could be correlated with alterations in the cytoskeleton, which leads to variations in the index of circularity of the nuclei and finally changes in cell morphology. These results are related to those reported by Ankam et al. [34], in which changes in cell nucleus sizes are associated with different types of cell pattern topographies.

### Quantification of fluorescence intensity: Vimentin

Vimentin is a cytoskeleton intermediate filament protein expressed in cells of mesenchymal origin. This provides architectural support and is essential in wound healing, but it can also promote excessive scarring [47]. Indirect immunofluorescence analysis showed an increase in the expression of vimentin as the incubation period progressed for each of the types of endomyocardial fibrosis evaluated, denoting changes in the intensity of the fluorescence expressed as fibroblast proliferation.

**Fig 13:**
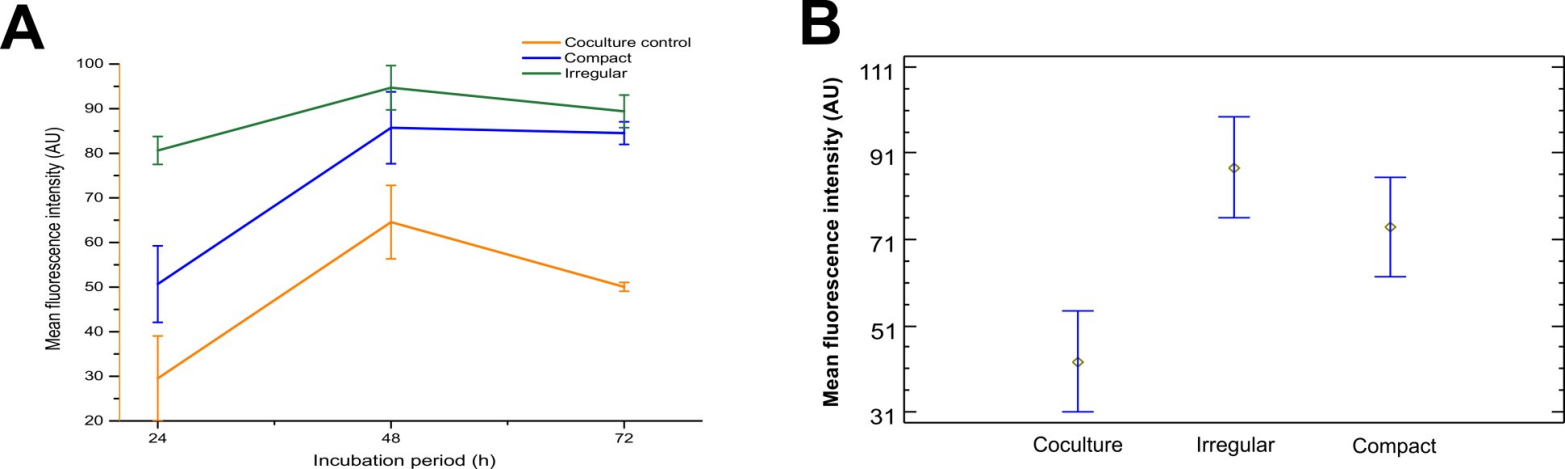
Results of average fluorescence at incubation period of 24, 48 and 72 h. (A) Mean fluorescence intensity in relation at incubation period. (B) Populations means of the fluorescence intensity in relation to the pattern type. The data are presented as mean ± standard deviation (n = 3).

The intensity values of each type of pattern were compared in relation to the coculture control in the incubation periods of 24, 48 and 72 h (**Fig 13**). During the 24 h incubation period, the average fluorescence intensity was 28% in the coculture control, 50.5% in the compact pattern, and 80.6% in the irregular pattern was observed. This behavior continued to increase and at the end of the 72 hours of incubation, the fluorescence intensity was higher for the irregular pattern (89.4%) compared to the compact pattern (84.5%). The values obtained for each incubation period showed statistically significant differences for each type of pattern compared to the coculture control, being 7% for the compact and 21% for the irregular **(Fig 13B).** On the other hand, the increase in fluorescence intensity expressed by vimentin in the irregular and compact pattern is related to the increase in the population density of fibroblasts as a possible consequence of an increase in the secretion of chemotactic factors such as TGF- β1, involved in fibrotic processes [28,48,49]. Likewise, the overexpression of the vimentin protein promotes invasive behavior and excessive scarring, leading to the formation of fibrotic focus, as a possible response to stress derived from organizational changes in tissue microarchitecture, which plays an important role in the cellular mechanics [47].

### Population count

Using the flow cytometry technique, cell proliferation of the population of RL-14 cardiomyocytes and 3T3 fibroblasts in compact and irregular EMF patterns in relation to coculture control was quantified, by CFSE labeling in cardiomyocytes. The growth rate of the two cell types at the incubation periods of 24, 48 and 72 h was determined. The spectrum of fluorescence intensity showed positive CFSE labeling (CFSE +) for cardiomyocytes and negative CFSE labeling (CFSE -) for fibroblasts.

**Fig 14:**
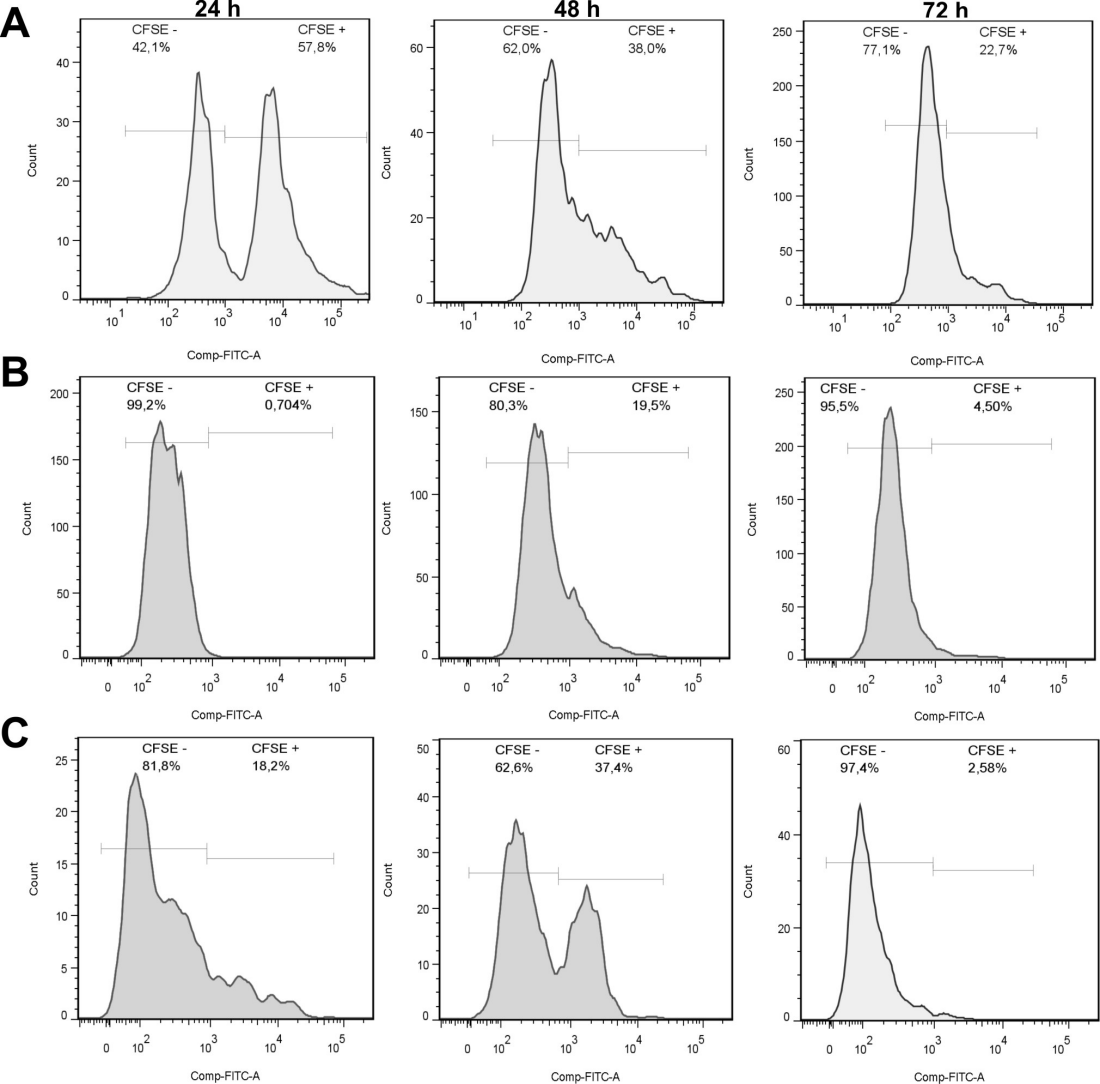
Cell proliferation in the interaction between RL-14 cardiomyocytes and 3T3 fibroblasts. (A) Coculture control at incubation periods of 24, 48 and 72 h. (B) compact pattern at incubation periods of 24 h, 48 h and 72 h. (C) irregular pattern at incubation periods of 24, 48 and 72 h. Positive labeling of CFSE for cardiomyocytes and negative labeling of CFSE for fibroblasts.

In the results presented in **Fig 14** for co-culture control, an increase in fibroblast proliferation and a decrease in cardiomyocytes were noted as incubation periods progressed. After 24 h the cell population corresponding to 3T3 fibroblasts for each type of pattern was 99.2% for the compact pattern (**Fig 14B**), 81.8% for the irregular one (**Fig 14C)** and 42.1% for the control coculture (**Fig 14A**). The cardiomyocyte cell population was 0.704% for the compact pattern, 18.2% for the irregular pattern and 57.8% for the coculture control. After 48 h of incubation, the population density of the fibroblasts increased in the coculture control by 19.9%, while in the compact and irregular pattern it decreased by 18.9% and 19.2%, respectively. On the other hand, the cardiomyocyte cell population increased by 18.8% in the compact pattern and 19.2% in the irregular pattern and decreased by 19.8% in the coculture control. At the end of the 72 h incubation period, a marked decrease in cardiomyocyte proliferation was shown, 15% for the compact pattern, 34.52% in the irregular pattern and 15.3% in the coculture control compared to the intensity of CFSE + detected at the 48 h of incubation, the opposite occurred with fibroblasts, which increased in all types of pattern, as well as in the coculture control, being 15.2% in the compact pattern, 34.8% in the irregular, and 15.1% in the coculture control. This behavior reflects the cellular loss of the population of cardiomyocytes in interaction with fibroblasts in structures that replicated EMF in patterns with compact and irregular distribution; these results are similar to those found in histopathological analyzes of the EMF, where the cardiomyocytes show a population decrease and the fibroblasts increase in the disposition of the scar tissue [42,50–52].

The platform developed in this study allowed to evaluate the cellular structure and interactions in EMF in an *in vitro* model using patterns with complex microarchitectures. When comparing the results obtained from this study with those reported from pathological samples of explanted EMF tissues [52], it was found that the irregular pattern characterized by a distribution of ramifications in the tissue geometry causes more tissue remodeling for the affected area and more cardiomyocyte morphological changes than the compact pattern, generating cells agglomerations [53]. In contrast, the compact pattern presented a lower index of tissue remodeling than the irregular pattern. This is because the compact pattern presents histopathologically dense areas of fibrosis formed by fibroblasts, which are related to those in a previous report Jeremias et al. [51]. However, the irregular pattern presents affected areas of cardiomyocytes intermingled with numerous fibroblasts.

In this investigation, an *in vitro* platform that emulates patterns of EMF was designed and developed, using Parafilm® inserts by means of the cell patterning technique. The results are consistent with those reported by Javaherian et al. [54,55], who used micro-patterning techniques that allowed the control of cellular and tissue architectures *in vitro* but whose study was limited to the generation of simple geometries. From this study, it is highlighted the limitations of the technique for the generation of complex domain microstructures.

*In vitro* models generating cellular functional units have been used to evaluate antiarrhythmic drugs, to develop biomaterials, and to implement new medical therapies. However, interactions of two or more types of cellular structures still is important to be validated. Based on others’ reports (including that by Zhao et al. who used only fibroblasts in their *in vitro* model of cardiac fibrosis) [37], and comparing those results with ours, our model is a potential platform for use in functional and therapeutic studies.

On the other hand, there are few in vitro models of cardiac fibrosis currently developed, which present differences between modeling techniques and the type of cells used. One of the investigations based on the development of an in vitro model of cardiac fibrosis was that of Spreeuwel et al [56], who based on the lithography technique imitated fibrotic behavior mediated by increases in population densities of fibroblasts and addition of concentrations of collagen, which demonstrated a negative effect on the contractile function of cardiomyocytes.Therefore, our study highlights some advantages over other in vitro models, because the distribution of the spatial distribution allows us to emulate EMF without the need for the introduction of additional components such as collagen, fibronectin, among others.

In summary, it was possible to reproduce specific microarchitectures of EMF and to determine the morpho-anatomical changes derived from the interaction between fibroblasts and cardiomyocytes. For future studies, it is expected to validate the generation of collagen associated with the variation in electrical activity in primary cardiac cells in an *in vitro* model of EMF replicated by the technique developed in this research.

## Conclusions

With the generation of cellular patterns using molds and the cell patterning technique, tissue patterns with specific spatial cell distributions, which favored the adhesion, proliferation, and generation of microdomains of cardiomyocytes and fibroblasts, were obtained.

Our results allowed to replicate EMF behaviors imitating the disruption of tissue, in which changes in the morphology of cardiomyocytes in interaction with fibroblasts were evidenced, mientras que, only a minority of the fibroblast population is usually affected due to its rapid proliferation.

In addition, the developed platform allowed to denote morphological changes in the cardiomyocytes in the presence of fibroblasts, from an elongated to a rounded shape and decrease in the cell density of the cardiomyocytes, likewise, cell loss of the myocytes was determined and an increase of cellular agglomerations derived from behaviors such as chemotaxis in the presence of 3T3 fibroblasts.

The modifications in the cell nuclei and the decrease in the population of the cardiomyocytes were caused by the direct interaction with fibroblasts, this behavior was modulated due to the microarchitectures of the patterns with complex geometries, which emulated characteristics of the EMF.

On the other hand, the results showed significant increases in cell proliferation of fibroblasts, responsible for generating support structures and increasing areas with a high density of collagen fibers during the development and evolution of endomyocardial fibrosis. This behavior inhibited the growth of cardiomyocytes, which showed a greater population decrease in irregular type patterns compared to the compact pattern.

## Supporting information

S1 Data (XLS)

S1 Fig Quantification of open areas. The red zone in segmentation indicates the open area. This is a image quantification process scheme using the Bio-EdIP software for segmentation and morphological reconstruction.
(TIF)

S2 Fig Compact pattern agglomerations. Compact EMF pattern at incubation period of 72 hours with the presence of agglomerates. 20× magnification. (Vimentin, red) and nuclei (hoechst, blue).
(TIF)

S3 Fig Irregular pattern agglomerations. Irregular EMF pattern at incubation period of 72 hours with the presence of agglomerates. 20× magnification. (Vimentin, red) and nuclei (hoechst, blue).
(TIF)
