# Supplementary figures and images for: Development of endomyocardial fibrosis model using a cell patterning technique: In vitro interaction of cell coculture of 3T3 fibroblasts and RL-14 cardiomyocytes
Source: PLoS One. 2020 Feb 24;15(2):e0229158. doi: 10.1371/journal.pone.0229158 (PMC7039516; doi:10.1371/journal.pone.0229158)

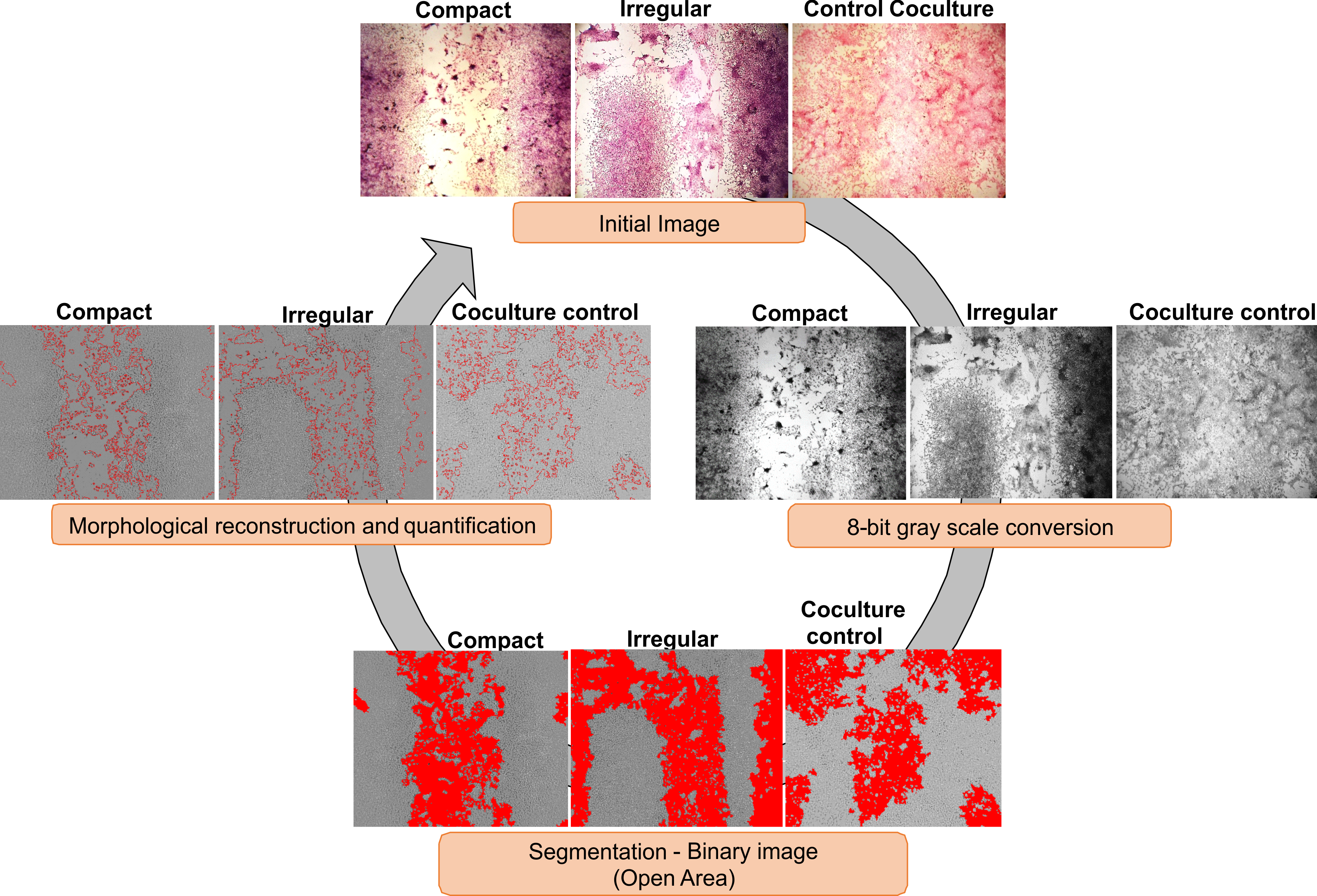

Supplement: S1 Fig — The red zone in segmentation indicates the open area. This is a image quantification process scheme using the Bio-EdIP software for segmentation and morphological reconstruction. (TIF) [file pone.0229158.s002.tif]

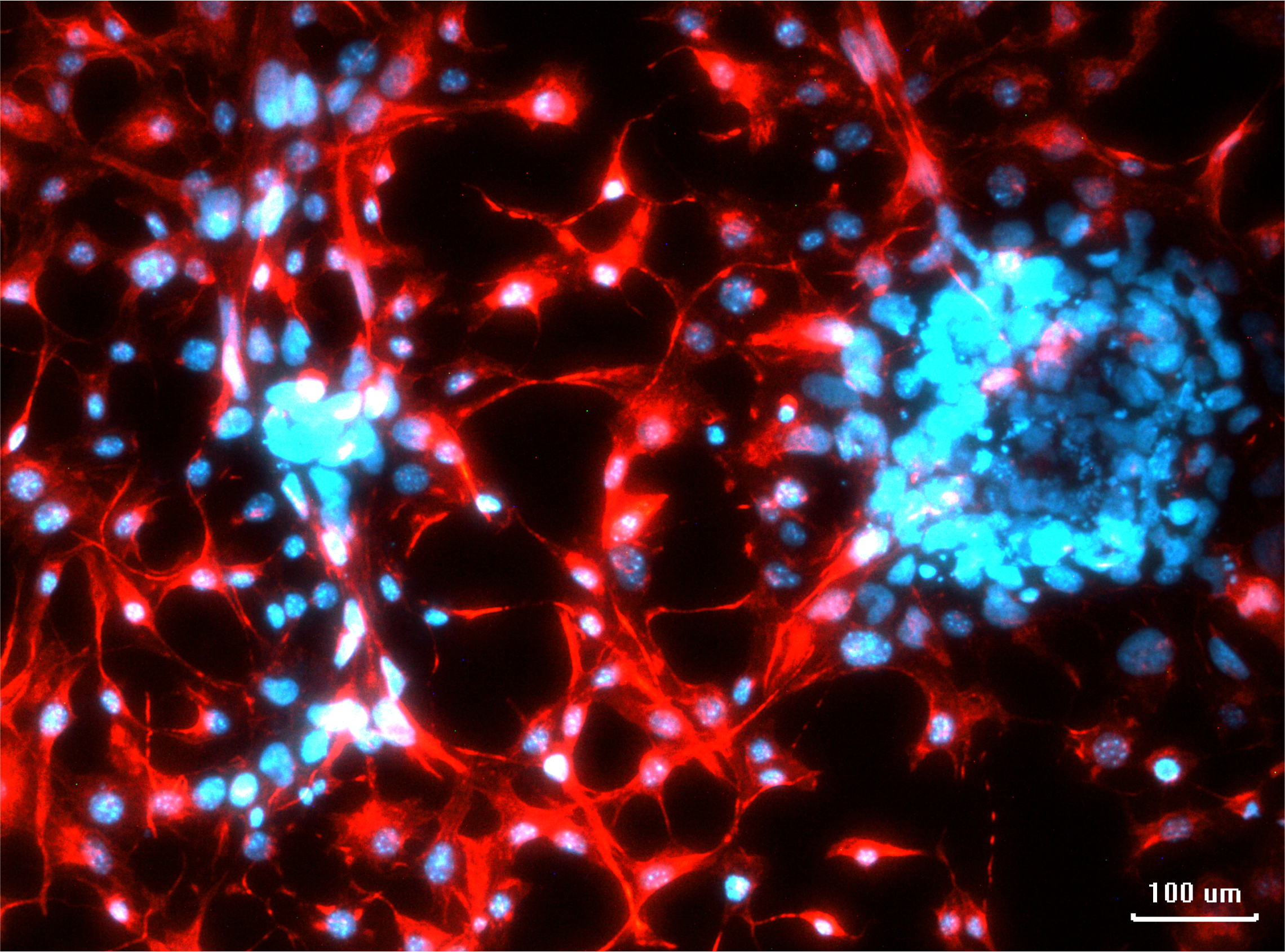

Supplement: S2 Fig — Compact EMF pattern at incubation period of 72 hours with the presence of agglomerates. 20× magnification. (Vimentin, red) and nuclei (hoechst, blue). (TIF) [file pone.0229158.s003.tif]

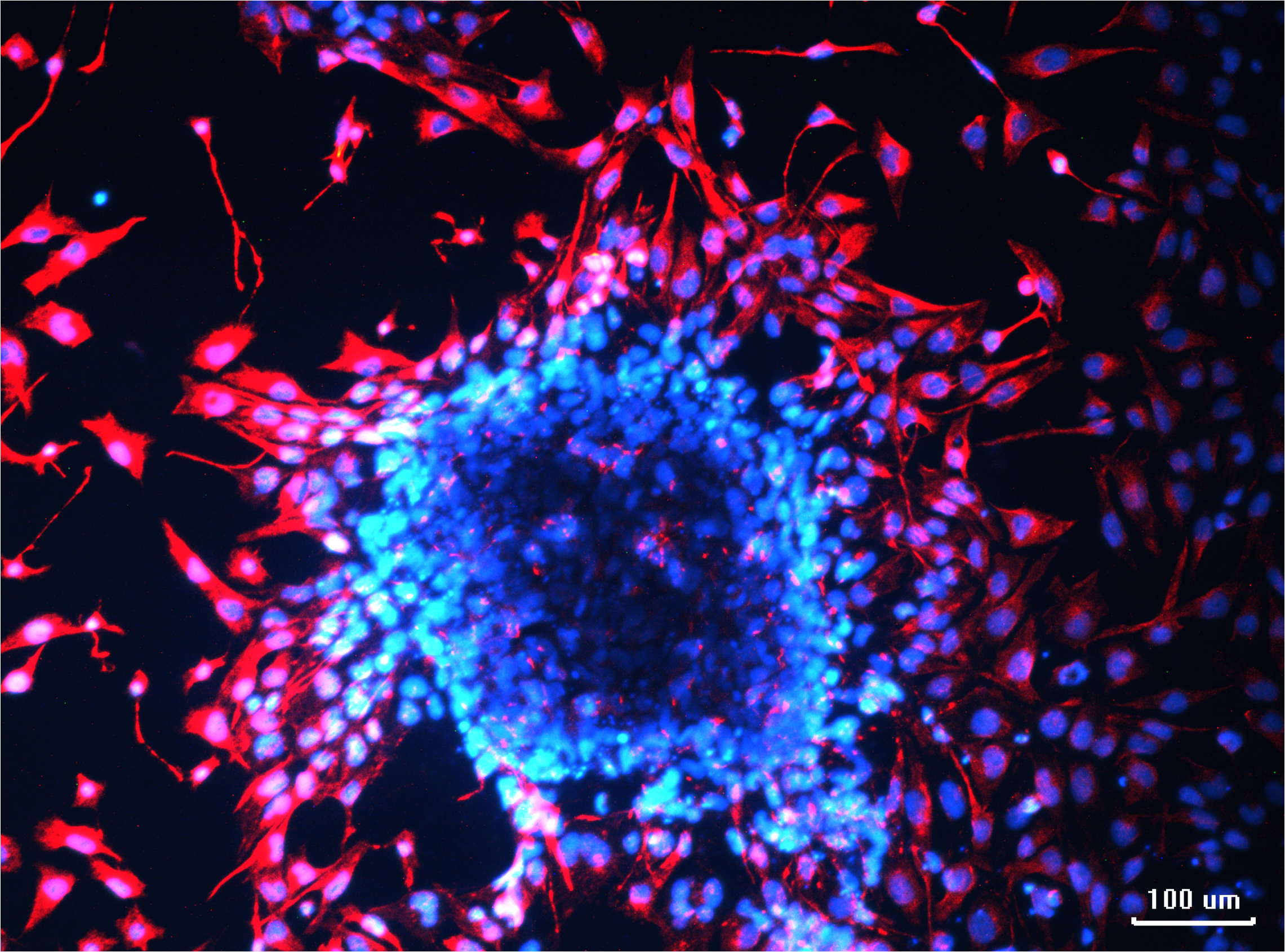

Supplement: S3 Fig — Irregular EMF pattern at incubation period of 72 hours with the presence of agglomerates. 20× magnification. (Vimentin, red) and nuclei (hoechst, blue). (TIF) [file pone.0229158.s004.tif]
